# Probiotic Characterization of *Lactiplantibacillus paraplantarum* SDN1.2 and Its Anti-Inflammatory Effect on *Klebsiella pneumoniae*-Infected Mammary Glands

**DOI:** 10.3390/vetsci12040323

**Published:** 2025-04-01

**Authors:** Jia Cheng, Jingdi Tong, Can Li, Ziyan Wang, Hao Li, Meiyi Ren, Jinshang Song, Deyuan Song, Qinna Xie, Mingchao Liu

**Affiliations:** Department of Clinical Veterinary Medicine, College of Veterinary Medicine, Hebei Agricultural University, Baoding 071001, China; tongjingdi@pgs.hebau.edu.cn (J.T.); 2022125010219@stu.hebau.edu.cn (C.L.); 20247201748@pgs.hebau.edu.cn (Z.W.); 20242200643@pgs.hebau.edu.cn (H.L.); renmeiyi@pgs.hebau.edu.cn (M.R.); 20232200614@pgs.hebau.edu.cn (D.S.); 20237201621@pgs.hebau.edu.cn (Q.X.)

**Keywords:** bovine mastitis, *Klebsiella pneumoniae*, *Lactiplantibacillus paraplantarum*, anti-inflammatory

## Abstract

*K. pneumoniae* is a major cause of bovine mastitis worldwide, making it difficult to control due to its resistance to multiple drugs. *L. paraplantarum* has been explored as a promising new approach to fighting bovine mastitis. In this study, the probiotic potential and safety of *L. paraplantarum* SDN1.2, as well as its ex vivo and in vivo anti-inflammatory effects against *K. pneumoniae*-induced mastitis, were comprehensively investigated using bioinformatics analyses and experimental validation methods. The results revealed that *L. paraplantarum* SDN1.2 exhibits non-hemolytic activity, is not cytotoxic, lacks virulence genes (e.g., adhesion factors, toxins, and invasion factors) and antibiotic resistance genes (e.g., beta-lactamases and tetracycline resistance genes), as supported by whole-genome sequencing, and significantly inhibits the growth of *K. pneumoniae*, as evaluated by antimicrobial tests. Following further validation in vitro, *L. paraplantarum* SDN1.2 demonstrated the capability to inhibit the adhesion and invasion of *K. pneumoniae* to bMECs. In a mouse model of *K. pneumoniae*-induced mastitis, *L. paraplantarum* SDN1.2 reduced the extent of neutrophil infiltration and inflammatory lesions. Furthermore, *L. paraplantarum* SDN1.2 pretreatment significantly reduced myeloperoxidase (MPO) activity and the expression of inflammatory cytokines (*IL-6*, *IL-1β*, and *TNF-a*) in mouse mammary gland tissue. In *K. pneumoniae*-infected bMECs, *L. paraplantarum* SDN1.2 significantly lowered lactate dehydrogenase (LDH) levels and expression of inflammatory cytokines such as *IL-6*, *IL-1β*, and *TNF-α*. The results demonstrated that the newly isolated *L. paraplantarum* SDN1.2 from bovine sources exhibits promising characteristics as a safe probiotic for the alleviation of bovine mastitis due to its safety profile and anti-inflammatory and antibacterial properties.

## Simple Summary

One of the most prevalent illnesses in dairy farms is mastitis. Although *Lactiplantibacillus paraplantarum* (*L. paraplantarum*), a significant probiotic with several uses, may have anti-inflammatory qualities, it remains unclear how it prevents mastitis. This study aimed to investigate the probiotic properties of *L. paraplantarum* SDN1.2 and its impact on mastitis caused by *Klebsiella pneumoniae* (*K. pneumoniae*) both in vitro and in vivo. Whole-genome sequencing analysis and in vitro tests confirmed that the application of *L. paraplantarum* SDN1.2 is safe and exhibits good probiotic properties. The results indicate that *L. paraplantarum* SDN1.2 mitigates *K. pneumoniae*-induced inflammation in mastitis, highlighting the therapeutic potential of *L. paraplantarum* in mastitis.

## 1. Introduction

Bovine mastitis, an infection of the mammary gland mostly caused by certain bacteria, poses a prevalent and challenging concern in dairy herds worldwide [1]. One important opportunistic bacterium that causes clinical mastitis is *K. pneumoniae* [2]. This bacterium, ubiquitously present in environmental reservoirs (e.g., soil and water), exhibits dual threats to both animal and human health through zoonotic potential and antimicrobial resistance dissemination [3]. Upon infection, an inflated immune response is frequently linked to *K. pneumoniae*-induced mastitis, which can result in severe clinical episodes, a marked decrease in milk output, and changes in inflammation of the udder [4]. In addition, *K. pneumoniae* rapidly adheres to and invades bovine mammary epithelial cells (bMECs) to evade antimicrobial therapy [5]. Furthermore, *K. pneumoniae* causes cell damage and apoptosis, including cell membrane rupture, nuclear membrane fragmentation, chromatin aggregation, mitochondrial swelling, and cell collapse, as well as an increase in concentrations of *TNF-α*, *IL-1β*, and *IL-8* in the mammary gland [6].

Antibiotics are typically used as a routine treatment for bovine mastitis [7]. However, antibiotic resistance among the pathogenic bacteria that cause bovine mastitis has significantly increased due to the overuse and improper application of antibiotics in dairy herds [8]. This has resulted in treatment failures that pose a serious threat to the health of dairy cows. In addition, antibiotic residues have been detected in cow milk following treatment, which can result in economic burdens on dairy farms when contaminated milk is discarded [9]. The overuse of antibiotics not only contributes to environmental pollution but also leads an increased risk of premature slaughter (i.e., the early culling of cows due to chronic mastitis-related complications, such as irreversible udder damage or systemic infections) [10]. Moreover, *K. pneumoniae* control is impaired by multidrug-resistant bacteria, which pose a global threat to dairy production [11].

Probiotics are generally recognized as safe (GRAS) microorganisms [12]. Probiotics’ most important strain properties include antioxidant activity, reduced expression of inflammatory factors, antibacterial activity against potential pathogenic bacteria or fungi, and the ability to reduce pathogen adherence to surfaces [13]. Several studies have reported that probiotics, especially *Lactobacillus* spp. have anti-inflammatory effects in vivo and in vitro. For example, *Lactobacillus plantarum* regulates the NF-κB signaling activation pathway, reducing related inflammatory cytokines in mastitis [14]. Yue et al. [15] found that *L. plantarum* could reduce the expression of TLR4, IL6, and TNFα as well as jejunal injury and had a protective effect against diarrhea caused by enterotoxigenic *Escherichia coli*. Thus, we hypothesized that *L. paraplantarum* might have a protective effect against inflammatory injury in mastitis cows. However, our analysis of the present literature reveals that related basic research is still limited.

In this study, the probiotic *L. paraplantarum* SDN1.2 with potential anti-inflammatory activity was selected. The study aimed to investigate the potential protective role of this probiotic against *K. pneumoniae*-induced inflammation in bMECs and mammary gland inflammatory responses in mice, and to form a basis to develop effective microecological preparations.

## 2. Materials and Methods

### 2.1. Bacterial Strains

*L. paraplantarum* SDN1.2 (GenBank accession number: PRJNA1233565) was isolated by our laboratory and preserved at the China General Microbiological Culture Collection Center (CCTCC; No. 28881). *L. paraplantarum* SDN1.2 was cultured in de Man, Rogosa, and Sharpe (MRS) (Hopebio, Qingdao, China) broth and incubated at 37 °C under anaerobic conditions for 24 h. Subsequently, sequence similarity was assessed using the BLAST tool (version 2.13.0; NCBI), and a phylogenetic tree was constructed using the neighbor-joining method in MEGA 7.0 software based on 16S rRNA gene sequences from 15 Lactobacillus strains. *K. pneumoniae* was provided by the laboratory of Mr. Gao Jian, School of Animal Medicine, China Agricultural University, China. *K. pneumoniae* was cultured in Brain Heart Infusion (BHI) broth (Hopebio, Qingdao, China) and incubated at 37  °C for 12  h with aerobic shaking.

### 2.2. DNA Extraction and Whole-Genome Sequencing

The EasyPure^®^ Bacteria Genomic DNA Kit (TransGen Biotech, Beijing, China) was used to extract the DNA from the host strain *L. paraplantarum* SDN1.2 according to the manufacturer’s instructions. The Nanodrop ND2000 spectrophotometer (ThermoFisher Scientific, Waltham, MA, USA) was used to evaluate the concentration and purity of the DNA, and electrophoresis on a 1% agarose gel was used to assess the quality of the DNA. The DNA library was constructed using the SQK-LSK109 ligation sequencing kit (Oxford Nanopore Technologies, Oxford, UK) to ensure that its concentration, purity, and integrity satisfied the requirements of the ensuing analysis. The library concentration was then measured using the Invitrogen Qubit 3.0 (Thermo Fisher Scientific, Waltham, MA, USA). The Illumina HiSeq™ 2000 sequencer (Illumina, San Diego, CA, USA) was used to sequence the final library. The predicted genes were aligned with multiple functional databases using BLAST (https://blast.ncbi.nlm.nih.gov/Blast.cgi accessed on 1 January 2020). Based on the highest BLAST score for each sequence, the alignment with the highest score (default identity ≥ 40%, coverage ≥ 40%) is annotated. The KEGG (Kyoto Encyclopedia of Genes and Genomes), eggNOG, CAZy (Carbohydrate-Active EnZymes Database), GO (Gene Ontology), and Pathogen–Host Interaction databases were annotated with the predicted coding genes.

### 2.3. Hemolytic Activity of L. paraplantarum SDN1.2

The hemolytic activity of *L. paraplantarum* SDN1.2 was assessed on blood agar supplemented with 5% (*v*/*v*) sheep blood (PB001, Land bridge, Beijing, China). Plates were streaked and incubated anaerobically at 37 °C for 24 h. *Staphylococcus aureus* served as the positive control. The hemolytic activity of the isolates was determined by the presence of β-hemolysis, as evidenced by a clear, colorless, or light-yellow zone surrounding the colonies.

### 2.4. Cytotoxicity Test

Cell viability was determined using Cell Counting Kit-8 (CCK-8; Solarbio Life Sciences, Beijing, China). The bMECs were seeded in 96-well plates (4 × 10^4^ cells per well) and cultured to 90% confluency at 5% CO_2_ and 37 °C under aerobic conditions. Cells were then treated with different concentrations of *L. paraplantarum* SDN1.2 (1 × 10^5^, 1 × 10^6^, 1 × 10^7^ CFU/mL) for 12 h. The untreated CONT group (BMECs without bacterial treatment) was included. After treatment, the medium was replaced with 100 μL of serum-free DMEM/F12, followed by the addition of 10 μL of CCK-8 solution. Thereafter, the cells were incubated at 37 °C for an additional 2 h. Absorbance was measured at 450 nm with a multimode microplate reader (Thermo Fisher Scientific, Waltham, MA, USA) to calculate cell viability.

### 2.5. Antibacterial Potential of L. paraplantarum SDN 1.2

The antibacterial activity of *L. paraplantarum* SDN1.2 was determined by the Oxford cup method [16] and the co-culture method [17]. For the Oxford cup method, *L. paraplantarum* SDN1.2 was anaerobically cultured in MRS broth at 37 °C for 24 h, centrifuged at 12,000 rpm for 20 min, and finally, the supernatant was filtered through a sterile filter with a pore size of 0.22 μm. A diluted *K. pneumoniae* culture (1:1000 dilution) was evenly spread on a Mueller–Hinton agar (MHA) (Hopebio, Qingdao, China) plate. Approximately 200 μL of *L. paraplantarum* SDN1.2 supernatant was added to each Oxford cup and incubated aerobically at 37 °C for 24 h using MRS medium (original pH 5.7) as a blank control, and the size of the inhibition zone was measured.

For the co-inoculated method, the concentrations of *L. paraplantarum* SDN1.2 and *K. pneumoniae* were adjusted to 1 × 10^8^ CFU/mL, and 100 μL of bacterial suspension of each of the two organisms was added together in 5 mL of MRS broth, while *K. pneumoniae* was used as a control, and incubated aerobically for 12 h and 24 h at 37 °C and 180 rpm, respectively. Viable counts of *K. pneumoniae* in the co-culture system were determined on the MacConkey inositol adonitol carbenicillin agar (MIAC) (Hopebio, Qingdao, China) plate.

### 2.6. Growth Curve of L. paraplantarum SDN1.2 in Different pH Levels

To evaluate the growth of *L. paraplantarum* SDN1.2 under different pH conditions, the pH of the MRS broth medium was adjusted from 4 to 7 (4.0, 4.5, 5.0, 5.5, 6.0, 6.5, and 7.0). The initial OD_600_ was measured immediately after inoculation of *L. paraplantarum* SDN1.2 into pH-adjusted MRS medium (approximately 0.2 at 0 h), and after 2, 4, 6, 8, 10, 12, 14, 16, 18, 20, 22, and 24 h of growth, the culture was vortexed to mix the cultures, and their absorbance at 600 nm was measured. MRS liquid medium not inoculated with *L. paraplantarum* SDN1.2 was selected as a blank control (OD_600_ value of approximately 0.17).

### 2.7. Antibacterial Activity Under Different pH Conditions

To investigate the antibacterial effects of *L. paraplantarum* SDN1.2 under different pH conditions, the MRS broth medium was adjusted to different pH values ranging from 4 to 7 (4, 4.5, 5.0, 5.5, 6.0, 6.5, and 7.0). *L. paraplantarum* SDN1.2 was inoculated in an MRS broth medium with different pH values. *K. pneumoniae* was adjusted to 0.5 McFarland (1 × 10^8^ CFU/mL), and 100 μL was plated on Mueller–Hinton agar (MHA). Next, 200 μL of cell-free supernatant was poured into the Oxford cups, while the untreated MRS broth medium (pH 5.7) served as a negative control. Following a 24-hour incubation period at 37 °C, the diameter of the inhibitory zones was measured.

### 2.8. Animal Experiments

The experimental study used SPF-grade Kunming mice from SPF (Beijing, China) Biotechnology Co., Ltd., that were around ten weeks old and weighed about fifty grams. Between three and seven days postpartum, a group of female mice were selected and divided into three groups: CONT, KP, and KP + *L. paraplantarum* SDN1.2 (*n* = 6 per group). After anesthesia using 2% Zoletil 50 (Sigma-Aldrich, St. Louis, MO, USA), the KP group as well as the KP + *L. paraplantarum* SDN1.2 group were injected with 100 μL of *K. pneumoniae* suspension (1 × 10^4^ CFU/mL), and the CONT group was injected with the same volume of PBS using a microsyringe through a nipple catheter; 100 μL of *L. paraplantarum* SDN1.2 bacterial suspension (1 × 10^6^ CFU/mL) was injected into the nipple of the KP + *L. paraplantarum* SDN1.2 group 24 h after the *K. pneumoniae* injection, and the same volume of PBS was injected into the KP group as well as the CONT group. Following a 24-h injection of *L. paraplantarum* SDN1.2, the mice were anesthetized and euthanized, and tissues from the mammary glands were gathered for additional examination. Hebei Agricultural University’s Experimental Animal Ethics Committee approved and allowed the procedures used on the animals in this study. The approval date was 15 June 2023, and the approval number is 2023089.

### 2.9. Histopathological Observations

Mammary glands of female mice were collected and fixed in a 4% formaldehyde solution. Mammary tissue was dehydrated in graded alcohol series, before being cleared in xylene and embedded in paraffin. Sections were stained with hematoxylin and eosin (H&E), and pathological and histological changes in the mammary glands were observed under a light microscope (100× and 400×). The extent of damage to mammary tissue was assessed using a scoring system based on a previous study [18]. Three independent analysts evaluating each section. The scoring system was based on the damage to the mammary tissue. There were five categories for each section: no impairment, mild impairment, moderate impairment, severe impairment, and very severe impairment, which are represented by scores 0–4.

### 2.10. Bacterial Load in the Mammary Glands

The number of *K. pneumoniae* colonies in mammary tissues was determined using a 10-fold serial dilution method to assess bacterial colonization. A tissue homogenizer was used to homogenize 0.1 g of the mammary gland in one milliliter of phosphate-buffered saline (PBS). The homogenate was then diluted using a 10-fold serial dilution procedure and incubated on MIAC plates at 37 °C for 12 h. The number of *K. pneumoniae* colonies was calculated.

### 2.11. Myeloperoxidase Evaluation

The mammary gland tissue was harvested and homogenized on ice with reaction buffer (weight/volume ratio 1:9). The detection method of MPO activity was carried out according to the manufacturer’s instructions (Nanjing Jiancheng Institute of Bioengineering, Nanjing, China). Enzyme activity was measured by measuring absorbance at 460 nm. (ODtest-ODcontrol)/[11.3 * weight (g)] is the MPO activity. The test used tissue homogenate, while the control used distilled water.

### 2.12. Cell Culture and Treatment

The cells were grown using the bovine mammary epithelial cell line MAC-T, which was acquired from the Shanghai Jingma Biological Technology (Shanghai, China). To maintain the cell culture, 89% DMEM/F12 medium (Procell, Wuhan, China) was mixed with 10% fetal bovine serum (FBS) (Meilun Bio, Dalian, China) and 1% penicillin–streptomycin solution (Solarbio, Beijing, China). bMECs were seeded in 6-well plates (8 × 10^5^ cells per well) or 96-well plates (4 × 10^4^ cells per well), grown to the logarithmic growth phase, and then incubated at 37 °C with 5% CO_2_. After achieving approximately 90% confluence by visual inspection, they were transferred to 6-well plates (approximately 2.4 × 10^6^ cells per well) or 96-well plates (approximately 2 × 10^5^ cells per well), and the cells were rinsed twice with phosphate-buffered saline (PBS) for subsequent experiments.

The cells were divided into four groups, each with three sample replicates: the control group (CONT), which did not receive any treatment; the *K. pneumoniae* group (KP), in which bMECs were infected with *K. pneumoniae* based on a 5:1 multiplicity of infection (MOI, or the ratio of *K. pneumoniae* to cells) for 6 h; the *L. paraplantarum* SDN1.2 group (*L. paraplantarum* SDN1.2), in which bMECs were infected with 1 × 10^6^ CFU/mL *L. paraplantarum* SDN1.2 for 9 h; and the *K. pneumoniae* + *L. paraplantarum* SDN1.2 group (KP + *L. paraplantarum* SDN1.2), in which bMECs were first pretreated with 1 × 10^6^ CFU/mL of *L. paraplantarum* SDN1.2 for 3 h, followed by infection with *K. pneumoniae* for 6 h.

### 2.13. Hematoxylin and Eosin Staining

The bMECs were seeded in 6-well plates with 8 × 10^5^ cells per well and grown to approximately 90% confluence. The bMECs were then pretreated with *L. paraplantarum* SDN1.2 for 3 h before adding *K. pneumoniae* and were then incubated at 37 °C with 5% CO_2_ for 3, 6, and 9 h. After being washed with PBS, the bMECs were fixed for 20 min in 4% paraformaldehyde, rinsed with PBS three times, and allowed to dry naturally for ten minutes. The manufacturer’s instructions were followed while performing hematoxylin–eosin (HE) (Beyotime Biotechnology, Shanghai, China) staining. Finally, cells in randomly selected fields were examined under an optical microscope (COIC, Chongqing, China) at 200× magnification.

### 2.14. Lactate Dehydrogenase (LDH) Release Assay LDH

The bMECs were seeded on 96-well plates and cultured until 90% confluency at 5% CO_2_ and 37 °C. The bMECs were pretreated with *L. paraplantarum* SDN1.2 for 3 h and/or infected with *K. pneumoniae* for 6 h. The supernatant from the bMECs was collected and measured using an LDH cytotoxicity assay kit (Beyotime Biotechnology Co., Ltd., Shanghai, China). A microplate reader was used to measure the absorbance value at 490 nm.

### 2.15. L. paraplantarum SDN1.2 Pretreatment on K. pneumoniae Adhesion and Invasion bMEC Assay Test

Adhesion and invasion of *K. pneumoniae* to bMECs were detected as previously described [5]. For the adhesion test, bMECs were inoculated into 6-well plates at 8 × 10^5^/well. The bMECs were cultured in 6-well plates until they reached about 90% confluence, washed three times with PBS, and the cells were pretreated for 3 h using DMEM/F12 basal medium with the concentration of *L. paraplantarum* SDN1.2 adjusted to 1 × 10^6^ CFU/mL, followed by the addition of *K. pneumoniae* according to the MOI = 5. *K. pneumoniae* co-interacted with bMECs for 3, 6, and 9 h. The *K. pneumoniae* alone treatment group was used as a positive control. The cells were then washed with PBS, and the cell suspension was doubly diluted and incubated on MIAC plates at 37 °C for 12 h. The number of *K. pneumoniae* colonies adhering to the surface of the bMECs was counted.

For the invasion test, bMECs were treated as described above. The bMECs were washed three times with PBS and treated with 50 µg/mL kanamycin per well for 2 h to kill extracellular *K. pneumoniae*. Then, 1 mL of 5% TritonX-100 (Solarbio) per well was added for 10 min to obtain the cell lysate, and the lysate was diluted in multiplicity and incubated on MIAC plates at 37 °C for 12 h. The number of *K. pneumoniae* bacteria invading the interior of the bMECs was counted by culture.

### 2.16. RNA Extraction and Real-Time PCR QPCR

Total RNA from bMECs and mouse mammary tissue was extracted using the TransZol Up Plus RNA kit (Quanshijin, Beijing, China). All RNA samples were subsequently reverse-transcribed into cDNA using a reverse transcription kit (Quanshijin, Beijing, China). Quantitative real-time RT-PCR was performed through using a CFX96 Real-Time PCR system (Bio-Rad, Hercules, CA, USA) with SYBR Green qPCR Master Mix (Quanshijin, Beijing, China). The mRNA expression of *TNF-α*, *IL-6*, and *IL-1β* was normalized to the mRNA expression of *GAPDH*. The primer sequences are given in Table 1. Using the instrument’s default melting curve acquisition software, the PCR amplification process involved predenaturing at 95 °C for 30 s, 95 °C for 10 s, 60 °C for 30 s, and 40 cycles. In this experiment, *GAPDH* was used as an internal reference gene, and the mRNA levels of the relevant target genes were calculated by the 2^−ΔΔCt^ method.

### 2.17. Enzyme-Linked Immunosorbent Assay (ELISA)

Mammary tissue was homogenized with phosphate-buffered saline (PBS) and then centrifuged at 12,000 r/min for 20 min to collect the supernatants. After treating the bMECs, the cell supernatants were collected as instructed. The levels of *IL-1β*, *TNF-α*, and *IL-6* in the mammary tissue and cells were quantified using a commercial ELISA kit (Shanghai Enzyme-linked Biotechnology Co., Ltd., Shanghai, China) following the manufacturer’s instructions. The absorbance was measured at 450 nm using a microplate reader.

### 2.18. Data Analysis

GraphPad Prism 8 was used for statistical analysis, which involved a one-way ANOVA with a significance level of *p* < 0.05. Results from all experiments are expressed as the mean ± SEM.

## 3. Results

### 3.1. Whole-Genome Analysis of L. paraplantarum SDN1.2

**Figure 1:**
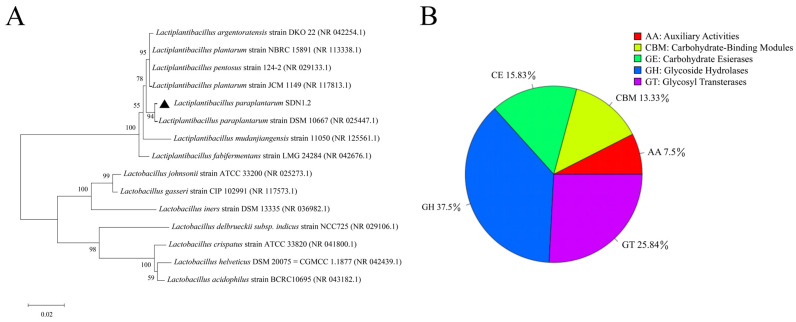
(**A**) phylogenetic tree. (**B**) Carbohydrate-active enzyme (CAZy) analysis.

The phylogenetic tree based on 16S rRNA sequences is shown in Figure 1A. The phylogenetic analysis indicated that SDN1.2 is closely related to the *Lactiplantibacillus paraplantarum* strain DSM 10667. Consequently, SDN1.2 was identified as *L. paraplantarum* and named *Lactiplantibacillus paraplantarum* SDN1.2.

**Figure 2:**
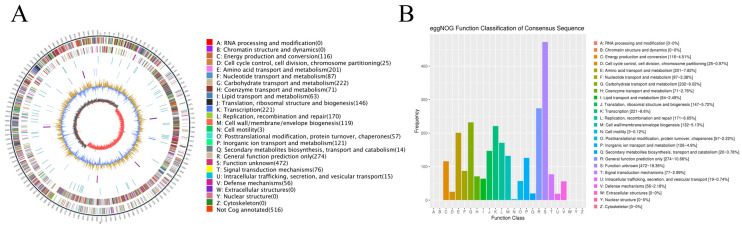
(**A**) A circular map of the chromosome. (**B**) Non-supervised orthologous groups (eggNOG) functional classification.

Whole-genome sequencing was performed to explore the potential of *L. paraplantarum* SDN1.2. Genomic DNA was extracted using the EasyPure^®^ Bacterial Genomic DNA Kit (TransGen Biotech, Beijing, China), with a concentration of 194.7 ng/μL and an A260/A280 ratio of 2.11, indicating high purity and minimal protein contamination (Supplementary Data S1). Figure 2A shows a complete circular genome map of *L. paraplantarum* SDN1.2, which comprises a single chromosome and a single plasmid. Table 2 summarizes the basic information on the genome of *L. paraplantarum* SDN1.2. The total length of the genome of *L. paraplantarum* SDN1.2 was 3,246,458 bp, with a GC content of 43.74%. The genome encodes 3045 genes, including 16 rRNAs (5S, 16S, 23S) and 69 tRNAs with an average length of 886 bp. These gene sequences span 2,699,214 bp, representing 83.14% of the total genome sequence. The KEGG, eggNOG, GO, and CAZy databases were used to analyze the gene functions of *L. paraplantarum* SDN1.2.

EggNOG annotation categorized the genes into 20 functional groups (Figure 2B). Carbohydrate transport and metabolism comprised 232 genes (9.02% of the annotated genes), which suggests that this host strain has a significant role in carbohydrate metabolism. A total of 132 genes (5.13% of all annotated genes) in the genome of *L. paraplantarum* SDN1.2 were annotated with cell wall/membrane/envelope biogenesis. These genes are likely involved in maintaining cell integrity and functionality, which may contribute to the strain’s ability to withstand environmental stresses. Fifty-six annotated genes were identified in the defense mechanism (2.18% of all annotated genes), indicating that the strain could resist the digestive tract environment and provide necessary conditions for stable colonization of bovine mammary epithelial cells.

Figure 1B displays the CAZy database annotation findings. The following five functions are annotated: carbohydrate esterases (CEs), glycoside hydrolases (GHs), glycosyl transferases (GTs), auxiliary activities (AAs), and carbohydrate-binding modules (CBMs). In that order, the percentage of functions with annotations is 37.5, 25.83, 15.83, 7.5, and 13.33%. The results indicate that the strain has a certain genetic basis and application potential in the degradation of carbohydrates such as cellulose and glycosides.

**Figure 3:**
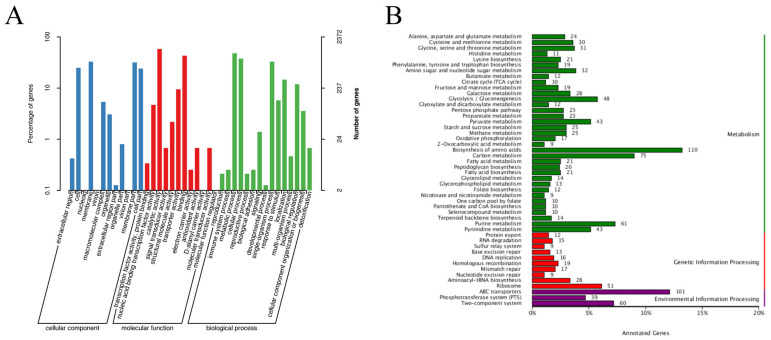
(**A**) Gene Ontology (GO) analysis. (**B**) Kyoto Encyclopedia of Genes and Genomes (KEGG) pathway enrichment.

The GO database classification system covers three important activities: biological processes, molecular functions, and cellular components (Figure 3A). The top five categories include membrane (one of the 12 categories of cellular components), metabolic processes and cellular processes (two of the 15 categories of biological processes), catalytic activity and binding (two of the 12 categories of molecular activities), and more. The results of cell component annotation indicate that *L. paraplantarum* SDN12 has strong biofilm formation ability, which may enhance bacterial protection against the external environment.

In total, 1484 genes were annotated in the KEGG database (Figure 3B) and classified into three major categories. There were 200, 189, and 905 annotated genes associated with environmental information processing, genetic information processing, and metabolism, respectively. The biosynthesis of amino acid pathway contained the highest number of genes, with 110 genes. ABC transporters and carbon metabolism followed this, and 101 and 75 genes were annotated, respectively. The above results indicate that *L. paraplantarum* SDN1.2 has a strong capacity for carbohydrate metabolism, amino acid metabolism, and membrane transport.

Furthermore, a large number of antimicrobials, anti-inflammatory, and immunoregulatory-related genes and their pathway information in the KEGG database have been annotated for *L. paraplantarum* SDN1.2, which is shown in Table 3. The genome of *L. paraplantarum* SDN1.2 contains genes related to antibiotic biosynthesis: monobactam biosynthesis, streptomycin biosynthesis, and antipathogenic defense mechanisms: peptidoglycan biosynthesis, ubiquinone and other terpenoid-quinone biosynthesis, and other antimicrobial-related genes, as well as genes involved in the regulation of inflammatory mediators: arachidonic acid metabolism, secondary bile acid biosynthesis, and antioxidant and cytoprotective properties: glutathione metabolism, taurine and hypotaurine metabolism, and other anti-inflammatory related genes. It also contains genes related to immune cell metabolism and signaling: purine metabolism, arginine and proline metabolism, and symbiotic bacterial-dormitory interactions: aminoacyl-tRNA biosynthesis, vitamin B6 metabolism, and other immunomodulation-related genes.

### 3.2. The Antibacterial Activity and Safety of L. paraplantarum SDN1.2

**Figure 4:**
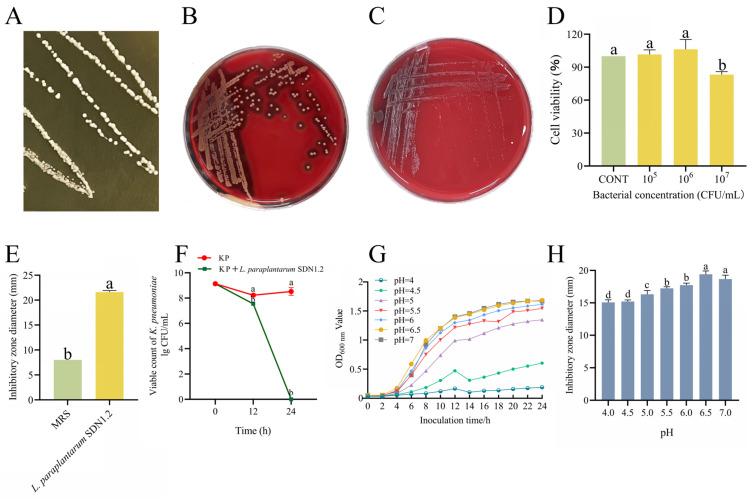
The growth characterization and antibacterial activity of *L. paraplantarum* SDN1.2. (**A**) Morphology of *L. paraplantarum* SDN1.2 on MRS agar medium. (**B**,**C**) Hemolytic test of *S. aureus* and *L. paraplantarum* SDN1.2. (**D**) Cytotoxicity test. (**E**) Antibacterial results of *L. paraplantarum* SDN1.2 and *K. pneumoniae* Oxford cup. (**F**) The results of co-culture of *L. paraplantarum* SDN1.2 and *K. pneumoniae* (KP). (**G**) Antimicrobial effect of *L. paraplantarum* SDN1.2 on *K. pneumoniae* under different pH conditions. (**H**) *L. paraplantarum* SDN1.2 growth curves under different pH conditions. The mean ± SEM was used for all data presentations. Different lowercase letters mean significant differences (*p* < 0.05).

**Figure 5:**
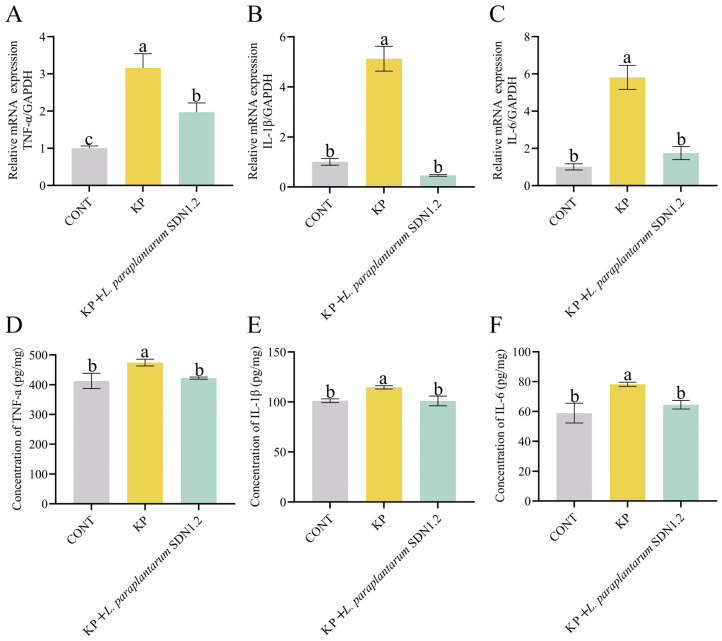
*L. paraplantarum* SDN1.2 attenuates *K. pneumoniae*-induced inflammation in mouse mastitis. (**A**–**C**) Mammary tissue homogenate’s *TNF-α*, *IL-1β*, and *IL-6* mRNA levels. (**D**–**F**) *TNF-α*, *IL-1β*, and *IL-6* protein levels in a homogenate of mammary tissue. The mean ± SEM was used for all data presentations. Different lowercase letters indicate significant differences (*p* < 0.05).

*L. paraplantarum* SDN1.2 forms small, smooth, milky round colonies in MRS agar medium (Figure 4A). In the hemolysis test, *S. aureus* showed a β-hemolytic zone (Figure 4B), while *L. paraplantarum* SDN1.2 had no hemolytic ring (Figure 4C), indicating *L. paraplantarum* SDN1.2 was non-hemolytic. The cytotoxicity assay results demonstrated that co-incubation of *L. paraplantarum* SDN1.2 at 1 × 10^6^ CFU/mL with bMECs for 12 h had no significant effect on cell viability (Figure 5D). The antibiotic resistance gene analysis of *L. paraplantarum* SDN1.2 was performed using CARD antibiotic resistance gene databases with identity >75%. Moreover, no resistance genes were annotated in *L. paraplantarum* SDN1.2.

Results of the Oxford cup experiment showed that *L. paraplantarum* SDN1.2 supernatant significantly inhibited the growth of *K. pneumoniae* (*p* < 0.05) (Figure 4E). *K. pneumoniae* counts in the co-culture group were significantly lower than the control group after both 12 and 24 h of co-cultivation (*p* < 0.05) (Figure 4F).

The growth curve of *L. paraplantarum* SDN1.2 at different pH levels is presented in Figure 4G. The growth performance of *L. paraplantarum* SDN1.2 is better at pH 6–7. With a continuous decrease in pH, the growth of *L. paraplantarum* SDN1.2 was inhibited to varying degrees. Figure 4H shows the antibacterial activity of *L. paraplantarum* SDN1.2 against *K. pneumoniae* under different pH conditions. Specifically, the maximum inhibitory diameter against *K. pneumoniae* is observed at pH 6.5.

### 3.3. L. paraplantarum SDN1.2 Ameliorates K. pneumoniae-Induced Injury to Mouse Mastitis

**Figure 6:**
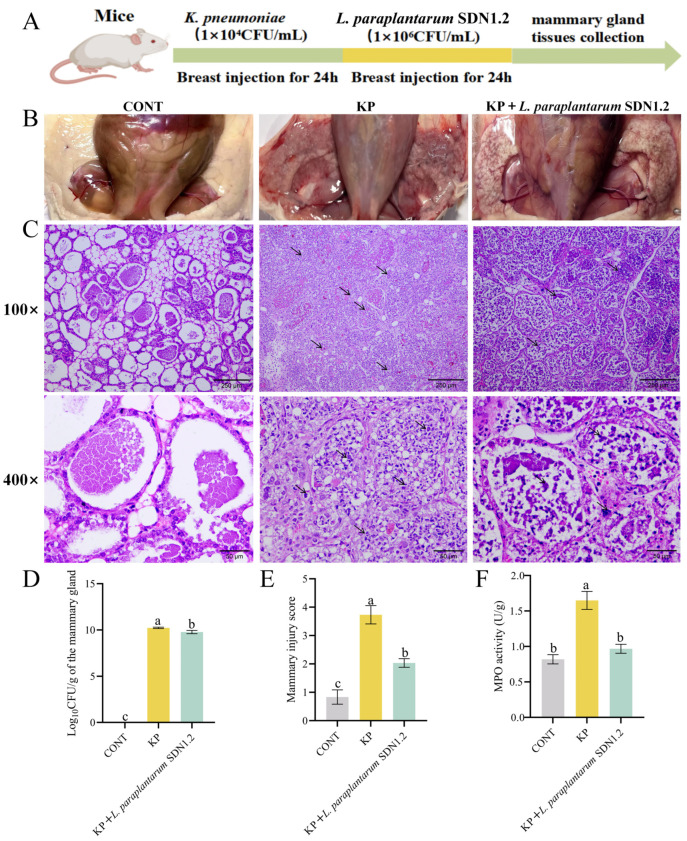
*L. paraplantarum* SDN1.2 ameliorates *K. pneumoniae*-induced injury to mouse mastitis. (**A**) Mouse therapeutic procedures. (**B**) Images of mouse mammary gland tissue from various treatment groups. (**C**) Mice in various treatment groups’ mammary gland tissue stained with HE. The arrows indicate the inflammatory cell infiltrates. (**D**) *K. pneumoniae* load in mammary tissue. (**E**) Mammary tissue histopathological score. (**F**) Mammary tissue MPO activity detection. The mean ± SEM was used for all data presentations. Different lowercase letters indicate significant differences (*p* < 0.05).

To confirm the effect of *L. paraplantarum* SDN1.2 on mastitis, we established a mouse mastitis model by injecting *K. pneumoniae* into the mammary ducts of mice (Figure 6A). Administration of *L. paraplantarum* SDN1.2 decreases the congestion and oedema of mammary tissue caused by *K. pneumoniae* (Figure 6B). The control group did not exhibit any bacterial colonization, as illustrated in Figure 6D. The *L. paraplantarum* SDN1.2 treatment groups had significantly lower bacterial loads in mammary tissue (*p* < 0.05) when compared to the KP group. H&E staining revealed that after the *K. pneumoniae* stimulation, infiltration of inflammatory cells and pathological damage were found in the mammary tissues. However, these changes were improved in the KP + *L. paraplantarum* SDN1.2 group, with a significant decrease in inflammation scores (*p* < 0.05) (Figure 6C,E). The MPO test also demonstrated that adding *L. paraplantarum* SDN1.2 may considerably lower the infiltration of inflammatory cells by *K. pneumoniae* (*p* < 0.05) (Figure 6F).

### 3.4. L. paraplantarum SDN1.2 Attenuates K. pneumoniae-Induced Inflammation in Mouse Mastitis

The mRNA expression levels of *IL-1β*, *IL-6*, and *TNF-α* in mammary tissues were measured using qRT–PCR. As shown in Figure 5A–C, the mRNA expression levels of *IL-1β*, *IL-6*, and *TNF-α* were higher in the KP group than in the CONT group (*p* < 0.05). The expression levels of *IL-1β*, *IL-6*, and *TNF-α* were lower in the KP + *L. paraplantarum* SDN1.2 group than in the KP group (*p* < 0.05).

*IL-1β*, *IL-6*, and *TNF-α* expression levels in mammary tissues were examined by ELISA. As shown in Figure 5D–F, the expression levels of *IL-1β*, *IL-6*, and *TNF-α* were higher in the KP group than in the CONT group (*p* < 0.05). The expression levels of *IL-1β*, *IL-6*, and *TNF-α* were lower in the KP + *L. paraplantarum* SDN1.2 group than in the KP group (*p* < 0.05). The ELISA and qRT–PCR results were consistent.

### 3.5. L. paraplantarum SDN1.2 Reduces the Cytotoxic Effects of bMECs in K. pneumoniae Infection

**Figure 7:**
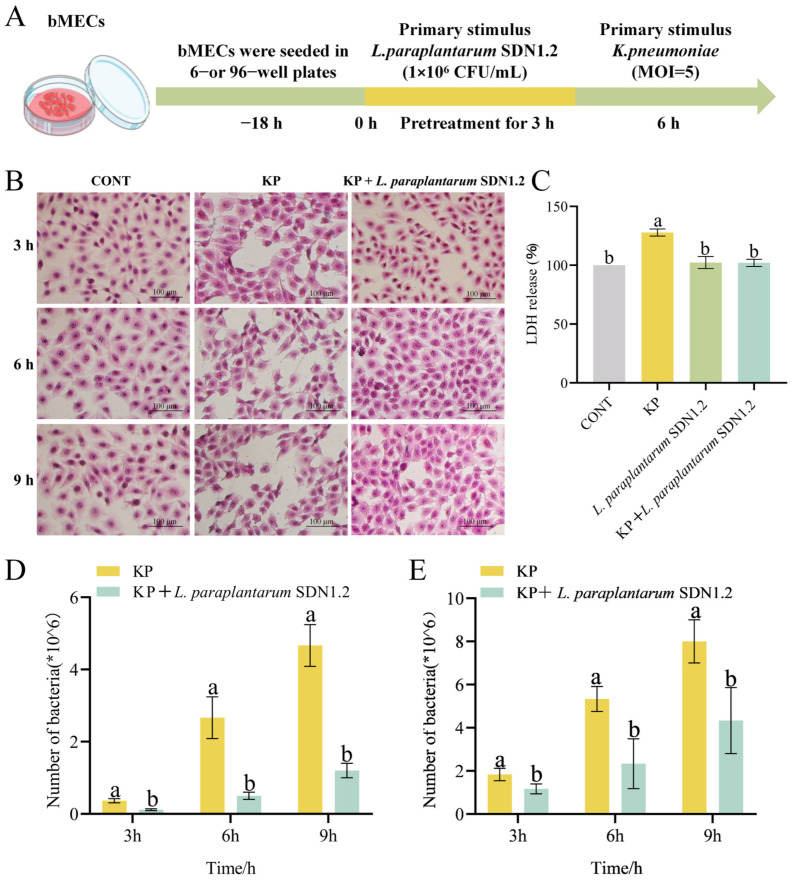
Pretreatment with *L. paraplantarum* SDN1.2 can improve bMEC damage in *K. pneumoniae* infection. (**A**) Cell culture and therapeutic procedures. (**B**) Morphological observations. (**C**) LDH release. (**D**,**E**) Adhesion (**D**) and invasion (**E**) of *K. pneumoniae*-infected bMECs in pretreatment of *L. paraplantarum* SDN1.2. The mean ± SEM was used for all data presentations. Different lowercase letters indicate significant differences (*p* < 0.05).

To evaluate the anti-inflammatory effects of *L. paraplantarum* SDN1.2 in vitro, we established a cellular mastitis model by challenging bMECs with *K. pneumoniae* (Figure 7A). Cell morphology and LDH assay were used to assess the cytotoxic effects of *K. pneumoniae* after incubation with *L. paraplantarum* SDN1.2 in bMECs. *K. pneumoniae*-infected bMECs exhibited morphological changes at 3 h post-infection (hpi), such as cellular enlargement (swelling), hyperchromatic nuclei (hyper-staining), and evidence of cell death (necrotic cell death). Furthermore, a reduction in bMEC number was observed. The extent of bMEC injury progressively increased with time. However, the group which had been pretreated with *L. paraplantarum* SDN1.2 showed significantly reduced swelling and necrosis in bMECs (Figure 7B). Regarding *K. pneumoniae*-infected bMECs, the KP group exhibited a significant increase in LDH release in comparison to the CONT group (*p* < 0.05), while the *L. paraplantarum* SDN1.2 group did not exhibit any significant change (*p* > 0.05). The LDH release of the SND1.2 + KP group was significantly lower in comparison to the KP group (*p* < 0.05) (Figure 7C).

### 3.6. L. paraplantarum SDN 1.2 Reduce Adhesion and Invasion of K. pneumoniae to bMECs

The finding of the current study showed that at three time points (3, 6, and 9 h), *L. paraplantarum* SDN1.2 significantly reduced the adhesion and invasion rate of *K. pneumoniae* in bMECs (*p* < 0.05) (Figure 7D,E).

### 3.7. L. paraplantarum SDN1.2 Inhibits the Inflammatory Response of bMECs Infected with K. pneumoniae

**Figure 8:**
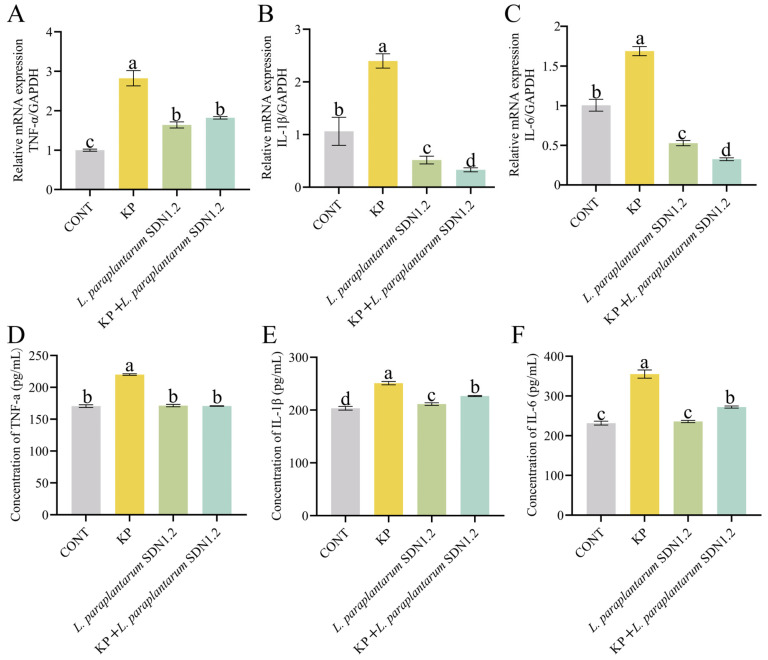
The bMECs infected with *K. pneumoniae* were prevented from releasing cytokines by *L. paraplantarum* SDN1.2. (**A**–**C**) Using GAPDH as the internal reference gene, RT-qPCR was utilized to assess the mRNA of *TNF-α*, *IL-1β*, and *IL-6*. (**D**–**F**) Levels of *TNF-α*, *IL-1β*, and *IL-6* were measured through ELISA. The mean ± SEM was used for all data presentations. Different lowercase letters indicate significant differences (*p* < 0.05).

*K. pneumoniae* substantially increased the mRNA expression levels of *IL-6*, *IL-1β*, and *TNF-α* (the KP group vs. the CONT group) in terms of anti-inflammatory potential (*p* < 0.05). On the other hand, the KP + *L. paraplantarum* SDN1.2 group, compared to the KP group, showed a significant decrease in the expression of these inflammatory markers (*p* < 0.05) (Figure 8A–C).

Following a *K. pneumoniae* infection, the supernatant’s levels of the inflammatory cytokines *TNF-α*, *IL-1β*, and *IL-6* were significantly elevated compared to those in the CONT group (*p* < 0.05). Comparing the cells with *L. paraplantarum* SDN1.2 before and after the treatment group to those with *K. pneumoniae*-infected bMECs, the amounts of the cytokines *TNF-α*, *IL-1β*, and *IL-6* were significantly lower (*p* < 0.05) (Figure 8D–F). According to the findings, *L. paraplantarum* SDN1.2 exerts anti-inflammatory effects in bMECs by reducing the expression of inflammatory factors produced by *K. pneumoniae*.

## 4. Discussion

*K. pneumoniae* is regarded as one of the principal causative agents of bovine mastitis, causing significant economic losses in the dairy sector and posing significant public health risks over the past decades [10]. The severe inflammation associated with *K. pneumoniae* mastitis is largely driven by the lipopolysaccharide (LPS) component of its cell wall. As a major constituent of the outer membrane of Gram-negative bacteria, LPS is known to trigger a robust immune response through recognition by Toll-like receptor 4 (TLR4) on host immune cells [19]. This interaction activates downstream signaling pathways, such as NF-κB and MAPK, leading to the production of pro-inflammatory cytokines (e.g., *TNF-α*, *IL-1β*, and *IL-6*) and chemokines [20]. In the context of mastitis, LPS from *K. pneumoniae* induces neutrophil infiltration, tissue damage, and systemic inflammatory responses, exacerbating the clinical manifestations of severe udder inflammation [21]. Furthermore, the structural variability of LPS, particularly the O-antigen, may influence the virulence and immune evasion strategies of *K. pneumoniae* strains, potentially affecting the severity of mastitis [22]. Currently, antibiotics are the primary therapies to treat *K. pneumoniae* mastitis. However, overuse of antibiotics and the slow development of novel antimicrobial agents are significant factors for the emergence of multidrug-resistant *K. pneumoniae* strains. Therefore, this critical situation demands urgent exploration of alternative treatment and prevention strategies. *L. paraplantarum* strains are gaining significant interest as potential antimicrobial alternatives due to their antimicrobial activity and immunomodulatory response [23]. To evaluate the potential of *L. paraplantarum* against *K. pneumoniae* infection, this study was designed. Our results demonstrate that *L. paraplantarum* SDN1.2 exhibited excellent biosafety, antibacterial activity, and anti-inflammatory properties against *K. pneumoniae*. However, the exact mechanism underlying the protective effects of *L. paraplantarum* SDN1.2 against *K. pneumoniae* mastitis requires further investigation.

Gene annotation enables the quick capture of relevant functional genes and safety data [24]. Thus, genome sequencing of *L. paraplantarum* SDN1.2 was carried out to assess its safety and anti-inflammatory potential using genes and genomes. Resistance assessment and hemolysis testing are significant strain screening parameters in the probiotic safety review procedure. The results of the testing revealed that *L. paraplantarum* SDN1.2 was not hemolytic and was not cytotoxic. Antibiotics are frequently used to kill invasive pathogens or inhibit their replication to control the spread of infection within the host. Antibiotic resistance ensures a probiotic’s survival within the host [25]. However, probiotics should be sensitive to at least two antibiotics or not carry intrinsic antimicrobial resistance genes, to reduce the risk of transmission. This investigation annotated target protein sequences using a BLAST-based CARD database based on whole-genome sequencing data from *L. paraplantarum* SDN1.2, and no resistance genes were identified. While drug susceptibility testing was not performed in the current study, our previous investigations demonstrated that *L. paraplantarum* SDN1.2 exhibited sensitivity to most antibiotics in standardized susceptibility assays [26]. Comprehensive hemolysis tests, drug sensitivity tests, and screening for resistance genes showed that the strain *L. paraplantarum* SDN1.2 was safe as a potential probiotic. In this work, the genome of *L. paraplantarum* SDN1.2 comprises genes encoding glycoside hydrolases (GHs) and glycosyl transferases (GTs). Glycoside hydrolases are catabolic enzymes of carbohydrate metabolic pathways, including glycosidases, which hydrolyze glycosidic bonds. The glycosidase glycosyl transferases are able to create glycosidic bonds from sugar donors having nucleoside phosphate or lipid phosphate leaving groups [27].

*L. paraplantarum* strains with antimicrobial properties generate significant interest in veterinary science and the livestock industry. Apart from its potential in veterinary medicine, *L. paraplantarum* has proven to be an important probiotic in human health. Previous studies have demonstrated that probiotics are effective in treating metabolic syndrome, diabetes, obesity, and gastrointestinal disorders [28]. It has been reported that *L. paraplantarum* 11 has broad-spectrum antimicrobial activity [29]. Our study shows that *L. paraplantarum* SDN1.2 exhibited excellent antibacterial activity against *K. pneumoniae*. Similarly, an already-reported investigation indicated that the cell-free supernatant of *L. plantarum* had a high antibacterial activity against numerous pathogenic enterobacteria (*E. coli*, *Shigella flexneri*, *Salmonella typhimurium*, *Proteus mirabilis*, and *Campylobacter jejuni*) [30]. Furthermore, *Lactobacillus* is known for its wide range of antimicrobial activities. It produces a variety of antimicrobial components, including organic acids, hydrogen peroxide, diacetyl, bacteriocins (plantaricins), and antimicrobial peptides, which are effective against a wide range of pathogenic microbes. Lactic acid bacteria are non-spore-forming, Gram-positive bacteria that can ferment sugar to produce a variety of organic acids [31]. A fall in pH can drastically reduce the development of other microorganisms. Furthermore, several investigations have indicated that H_2_O_2_, created during the metabolic process, can suppress microorganisms [32]. Therefore, we concluded that *L. paraplantarum* SDN1.2 may exert antimicrobial effects by producing some organic acids. Microorganisms produce specific proteins called secreted proteins during their life. These proteins can kill other organisms, enhancing environmental survival capabilities [33]. The host genomic sequence analysis showed that *L. paraplantarum* SDN1.2 84 potentially secreted proteins. These proteins may play a role in bacterium interactions with its environment, and some might have antimicrobial properties. Therefore, our future study objective is to determine the contribution of these secreted proteins to *L. paraplantarum*’s antimicrobial effects.

Controlling bacterial infections requires minimizing bacterial colonization. In this investigation, we discovered that udder tissues were highly colonized by bacteria, which is consistent with prior findings [34]. Supplementation with *L. paraplantarum* SDN1.2 effectively reduced bacterial colonization. Histopathological examination showed extensive infiltration of inflammatory cells and necrotic detached mammary epithelial cells in severely damaged mammary tissue. However, treatment with *L. paraplantarum* SDN1.2 decreased inflammatory cell infiltration and tissue damage. MPO is a marker for neutrophil invasion [35]. MPO levels in mammary tissue rose following *K. pneumoniae* infection, whereas supplementation with *L. paraplantarum* SDN1.2 significantly reduced MPO activity. As a result, supplementation with *L. paraplantarum* SDN1.2 can diminish inflammatory cell infiltration, hence alleviating the inflammatory response.

bMECs play a vital role in innate immunity, secreting cytokines like *IL-1β*, IL-6, and TNF-α, as well as enzymes like *iNOS* and *COX-2* during mammary gland inflammation. This release supports the recruitment of immune cells, such as neutrophils and macrophages, for pathogenic microorganism elimination [36]. Maintaining proper amounts of inflammatory cytokines is critical for an efficient immune response against infections; however, excessive production exacerbates inflammation and worsens udder damage [37]. Consequently, limiting the excessive release of inflammatory mediators during mammary gland inflammation is critical. Our findings show that *L. paraplantarum* SDN1.2 effectively reduces the release of these mediators both in vivo and in vitro, thereby reducing *K. pneumoniae*-induced mastitis. This anti-inflammatory effect is consistent with previous studies on lactic acid bacteria strains. For instance, *L. paraplantarum* CRL 2051 reduces the production of pro-inflammatory cytokines such as *TNF-α* and *IL-6* in a mouse model of metabolic disorders, highlighting its potential as an immunomodulator [38]. In the context of mastitis, *L. plantarum* KLDS 1.0344 has been reported to mitigate Escherichia coli-induced inflammation in bovine mammary epithelial cells by downregulating NF-κB-mediated signaling [14]. Additionally, the inhibition of *L. paraplantarum* SDN1.2-mediated inflammatory response was associated with the protection of bMECs from cytotoxicity. Lactate dehydrogenase (LDH) is a key indicator of bacterial cytotoxicity [39]. In this study, *K. pneumoniae* infection significantly increased LDH release from bMECs, indicating cellular damage and intracellular enzyme leakage. However, preincubation with *L. paraplantarum* SDN1.2 substantially reduced the elevated LDH release to normal levels, suggesting its protective effect against *K. pneumoniae*-induced cytotoxicity. This finding aligns with previous reports that *L. plantarum* strains can enhance epithelial barrier function and reduce cellular damage in inflammatory conditions [40].

Although probiotics have been proposed as alternatives in treating bovine mastitis, their limitations are well documented. Studies have shown that probiotics provide temporary protection for the breast by reducing the risk of infection and the severity of inflammation but do not achieve a cure for mastitis [41,42]. This transient efficacy highlights their role as a potential adjunct rather than a stand-alone solution for treating mastitis. However, probiotics might serve as an alternative to vaccination trials. Notably, probiotics may serve as a temporary alternative to vaccines in clinical trials but face challenges in inducing long-term adaptive immunity against multiple *K. pneumoniae* strains [43]. Despite these challenges, their capacity to transiently modulate immune responses and reduce acute infection highlights their potential as complementary tools in mastitis management.

## 5. Conclusions

In this study, we investigated the probiotic properties and anti-inflammatory capacity of *L. paraplantarum* SDN1.2. Through whole-genome sequencing analysis combined with in vitro experiments, we confirmed the biosafety of this strain and demonstrated an antimicrobial capacity. Furthermore, *L. paraplantarum* SDN1.2 reduced both the adhesion and invasion of *K. pneumoniae* in both in vitro and in vivo assays, and it attenuated *K. pneumoniae*-induced inflammatory responses by inhibiting the expression of inflammatory factors. This study provides evidence that *L. paraplantarum* ameliorates mastitis pathology and establishes a foundation for its potential application as a potential mastitis prevention strategy.

## Figures and Tables

**Table 1:** List of primers for real-time PCR.

Gene	Primer Sequence (5′-3′)		Product Length (bp)	Annealing Temperature (°C)	Accession Number
Bos					
*IL-6*	Forward	GCTGAATCTTCCAAAAATGGAGG	215	60	NM_173923.2
	Reverse	GCTTCAGGATCTGGATCAGTG			
*IL-1β*	Forward	CCTCGGTTCCATGGGAGATG	119	60	NM_174093.1
	Reverse	AGGCACTGTTCCTCAGCTTC			
*TNF-α*	Forward	TCCAGAAGTTGCTTGTGCCT	144	60	NM_173966.3
	Reverse	CAGAGGGCTGTTGATGGAGG			
*GAPDH*	Forward	GTCTTCACTACCATGGAGAAGG	201	60	NM_001034034.2
	Reverse	TCATGGATGACCTTGGCCAG			
Mice					
*IL-1β*	Forward	CCTGGGCTGTCCTGATGAGAG	188	60	NM_008361.4
	Reverse	TCCACGGGAAAGACACAGGTA			
*IL-6*	Forward	TAGTCCTTCCTACCCCAATTTCC	142	60	NM_001314054.1
	Reverse	TTGGTCCTTAGCCACTCCTTC			
*TNF-α*	Forward	CAGGCGGTGCCTATGTCTC	155	60	NM_001278601.1
	Reverse	CGATCACCCCGAAGTTCAGTAG			
*GAPDH*	Forward	AGGTCGGTGTGAACGGATTTG	139	60	NM_001289726.2
	Reverse	TGTAGACCATGTAGTTGAGGTCA			

**Table 2:** General genome features of *L. paraplantarum* SDN1.2.

Features	Results	Features	Results
Genome size	3,246,458	5S rRNA	6
GC content	43.74	16S rRNA	5
Number of genes	3045	23S rRNA	5
Total gene length	2,699,214	tRNA	69
Proportion of coding genes	83.14	eggNOG	2529
Mean gene length	886	GO	2372
Repeat sequence length	2813	KEGG	1484
Repeat sequence content	0.09	VFDB	0

**Table 3:** *L. paraplantarum* SDN1.2 genome’s antibacterial and anti-inflammatory pathway and related genes.

No	Pathway ID	Description	Gene Number
1	ko00261	Monobactam biosynthesis	7
2	ko00521	Streptomycin biosynthesis	4
3	ko00550	Peptidoglycan biosynthesis	20
4	ko00130	Ubiquinone and other terpenoid-quinone biosynthesis	6
5	ko00590	Arachidonic acid metabolism	1
6	ko00121	GOSecondary bile acid biosynthesis	2
7	ko00480	Glutathione metabolism	9
8	ko00430	Taurine and hypotaurine metabolism	5
9	ko00230	Purine metabolism	61
10	ko00330	Arginine and proline metabolism	6
11	ko00970	Aminoacyl-tRNA biosynthesis	28
12	ko00750	Vitamin B6 metabolism	3

## Data Availability

The data presented in this study are contained within the article.

## Supplementary Materials

The following supporting information can be downloaded at: https://www.mdpi.com/article/10.3390/vetsci12040323/s1, Supplementary Data S1: PRISMA 2020 Checklist; Supplementary Data S2: Nanodrop curve. Reference [44] is cited in Supplementary Materials.

## References

[1] Ashraf A., Imran M. (2020). Causes, types, etiological agents, prevalence, diagnosis, treatment, prevention, effects on human health and future aspects of bovine mastitis. Anim. Health Res. Rev..

[2] Schukken Y., Chuff M., Moroni P., Gurjar A., Santisteban C., Welcome F., Zadoks R. (2020). The “other” gram-negative bacteria in mastitis: *Klebsiella*, *Serratia*, and more. Vet. Clin. N. Am. Food Anim. Pract..

[3] Hu Y., Anes J., Devineau S., Fanning S. (2021). *Klebsiella pneumoniae*: Prevalence, Reservoirs, Antimicrobial Resistance, Pathogenicity, and Infection: A Hitherto Unrecognized Zoonotic Bacterium. Foodborne Pathog. Dis..

[4] Fuenzalida M.J., Ruegg P.L. (2019). Negatively-controlled, randomized clinical trial to evaluate intramammary treatment of nonsevere, gramnegative clinical mastitis. J. Dairy Sci..

[5] Yang J., Xiong Y., Barkema H.W., Tong X., Lin Y., Deng Z., Kastelic J.P., Nobrega D.B., Wang Y., Han B. (2024). Comparative genomic analyses of *Klebsiella pneumoniae* K57 capsule serotypes isolated from bovine mastitis in China. J. Dairy Sci..

[6] Cheng J., Zhang J., Han B., Barkema H.W., Cobo E.R., Kastelic J.P., Zhou M., Shi Y., Wang J., Yang R. (2020). *Klebsiella pneumoniae* isolated from bovine mastitis is cytopathogenic for bovine mammary epithelial cells. J. Dairy Sci..

[7] Angelopoulou A., Warda A.K., Hill C., Ross R.P. (2019). Non-antibiotic microbial solutions for bovine mastitis—Live biotherapeutics, bacteriophage, and phage lysins. Crit. Rev. Microbiol..

[8] Pal C., Bengtsson-Palme J., Kristiansson E., Larsson D.G. (2016). The structure and diversity of human, animal and environmental resistomes. Microbiome.

[9] Leite de Campos J., Gonçalves J.L., Kates A., Steinberger A., Sethi A., Suen G., Shutske J., Safdar N., Goldberg T., Ruegg P.L. (2023). Variation in partial direct costs of treating clinical mastitis among 37 Wisconsin dairy farms. J. Dairy Sci..

[10] Ziesch M., Wente N., Zhang Y., Zaremba W., Engl S., Kromker V. (2018). Noninferiority Trial Investigating the Efficacy of a Nonantibiotic Intramammary Therapy in the Treatment of Mild-to-Moderate Clinical Mastitis in Dairy Cows With Longer Lasting Udder Diseases. J. Vet. Pharmacol. Ther..

[11] Hoque M.N., Moyna Z., Faisal G.M., Das Z.C., Islam T., Newton I.L.G. (2023). Whole-genome sequence of multidrug-resistant *Klebsiella pneumoniae* MNH_G2C5, isolated from bovine clinical mastitis milk. Microbiol. Resour. Announc..

[12] Pineiro M., Stanton C. (2007). Probiotic bacteria: Legislative framework—Requirements to evidence basis. J. Nutr..

[13] Burkholder K.M., Fletcher D.H., Gileau L., Kandolo A. (2019). Lactic acid bacteria decrease *Salmonella enterica* Javiana virulence and modulate host inflammation during infection of an intestinal epithelial cell line. Pathog. Dis..

[14] Chen Q., Wang S., Guo J., Xie Q., Evivie S.E., Song Y., Li B., Huo G. (2021). The Protective Effects of *Lactobacillus plantarum* KLDS 1.0344 on LPS-Induced Mastitis In Vitro and In Vivo. Front. Immunol..

[15] Yue Y., He Z., Zhou Y., Ross R.P., Stanton C., Zhao J., Zhang H., Yang B., Chen W. (2020). *Lactobacillus plantarum* relieves diarrhea caused by enterotoxin-producing Escherichia coli through inflammation modulation and gut microbiota regulation. Food Funct..

[16] Zhang H., HuangFu H., Wang X., Zhao S., Liu Y., Lv H., Qin G., Tan Z. (2021). Activity of Lactic Acid Producing *Leuconostoc mesenteroides* QZ1178 Against Pathogenic *Gallibacterium anatis*. Front. Vet. Sci..

[17] Tsai C.C., Lai T.M., Hsieh Y.M. (2019). Evaluation of Lactobacilli for Antagonistic Activity Against the Growth, Adhesion and Invasion of *Klebsiella pneumoniae* and *Gardnerella vaginalis*. Indian J. Microbiol..

[18] Guo W., Liu B., Hu G., Kan X., Li Y., Gong Q., Xu D., Ma H., Cao Y., Huang B. (2019). Vanillin protects the blood-milk barrier and inhibits the inflammatory response in LPS-induced mastitis in mice. Toxicol. Appl. Pharmacol..

[19] Park B.S., Lee J.O. (2013). Recognition of lipopolysaccharide pattern by TLR4 complexes. Immunol. Rev..

[20] Liu Y., Chen W., Zheng F., Yu H., Wei K. (2022). Xanthatin Alleviates LPS-Induced Inflammatory Response in RAW264.7 Macrophages by Inhibiting NF-κB, MAPK and STATs Activation. Molecules.

[21] Burvenich C., Van Merris V., Mehrzad J., Diez-Fraile A., Duchateau L. (2003). Severity of E. coli mastitis is mainly determined by cow factors. Vet. Res..

[22] Singh S., Wilksch J.J., Dunstan R.A., Mularski A., Wang N., Hocking D., Jebeli L., Cao H., Clements A., Jenney A.W.J. (2022). LPS O Antigen Plays a Key Role in *Klebsiella pneumoniae* Capsule Retention. Microbiol. Spectr..

[23] Li K., Yang M., Tian M., Jia L., Du J., Wu Y., Li L., Yuan L., Ma Y. (2022). *Lactobacillus plantarum* 17-5 attenuates *Escherichia coli*-induced inflammatory responses via inhibiting the activation of the NF-κB and MAPK signalling pathways in bovine mammary epithelial cells. BMC Vet. Res..

[24] Chen Y., Chen M., Jin J., Bai Y., Wang Q., Liao Z., Chen Q. (2020). Isolation, identification, and whole genome sequence analysis of the alginate-degrading bacterium *Cobetia* sp. cqz5-12. Sci. Rep..

[25] Holzapfel W., Arini A., Aeschbacher M., Coppolecchia R., Pot B. (2018). *Enterococcus faecium* SF68 as a model for efficacy and safety evaluation of pharmaceutical probiotics. Benef. Microbes..

[26] Jin T., Feng M., Liang H., Liu K., Tong J., Ren M., Jiang G., Gao J., Liu M., Cheng J. (2025). Isolation and Identification of 3 Lactic Acid Bacteria and Their Antibacterial Activity against *Klebsiella pneumoniae* Isolated from Bovine Mastitis. Chin. J. Vet. Med..

[27] Cuyvers S., Dornez E., Delcour J.A., Courtin C.M. (2012). Occurrence and functional significance of secondary carbohydrate binding sites in glycoside hydrolases. Crit. Rev. Biotechnol..

[28] Liu Y.W., Liong M.T., Tsai Y.C. (2018). New perspectives of *Lactobacillus plantarum* as a probiotic: The gut-heart-brain axis. J. Microbiol..

[29] Kalhoro M.S., Anal A.K., Kalhoro D.H., Hussain T., Murtaza G., Mangi M.H. (2023). Antimicrobial Activities and Biopreservation Potential of Lactic Acid Bacteria (LAB) from Raw Buffalo (*Bubalus bubalis*) Milk. Oxidative Med. Cell. Longev..

[30] Danilova T.A., Adzhieva A.A., Mezentseva M.V., Suetina I.A., Danilina G.A., Minko A.G., Dmitrieva M.L., Zhukhovitsky V.G. (2023). The inhibitory activity of *Lactobacillus plantarum* supernatant against Enterobacteria, Campylobacter, and tumor cells. Bull. Exp. Biol. Med..

[31] Punia B.S., Suri S., Trif M., Ozogul F. (2022). Organic acids production from lactic acid bacteria: A preservation approach. Food Biosci..

[32] Pino A., Vaccalluzzo A., Caggia C., Balzaretti S., Vanella L., Sorrenti V., Ronkainen A., Satokari R., Randazzo C.L. (2022). *Lacticaseibacillus rhamnosus* CA15 (DSM 33960) as a Candidate Probiotic Strain for Human Health. Nutrients.

[33] Chernyatina A.A., Low H.H. (2019). Core architecture of a bacterial type II secretion system. Nat. Commun..

[34] Qiu M., Feng L., Yu Z., Zhao C., Gao S., Bao L., Zhang N., Fu Y., Hu X. (2022). Probiotic Enterococcus mundtii H81 inhibits the NF-κB signaling pathway to ameliorate Staphylococcus aureus-induced mastitis in mice. Microb. Pathog..

[35] Lin W., Chen H., Chen X., Guo C. (2024). The Roles of Neutrophil-Derived Myeloperoxidase (MPO) in Diseases: The New Progress. Antioxidants.

[36] Sumaiya K., Langford D., Natarajaseenivasan K., Shanmughapriya S. (2022). Macrophage migration inhibitory factor (MIF): A multifaceted cytokine regulated by genetic and physiological strategies. Pharmacol. Ther..

[37] Bhol N.K., Bhanjadeo M.M., Singh A.K., Dash U.C., Ojha R.R., Majhi S., Duttaroy A.K., Jena A.B. (2024). The interplay between cytokines, inflammation, and antioxidants: Mechanistic insights and therapeutic potentials of various antioxidants and anti-cytokine compounds. Biomed. Pharmacother..

[38] Isas A.S., Balcells M.F., Maldonado Galdeano C., Palomo I., Rodriguez L., Fuentes E., Luna Pizarro P., Mateos Briz R., Mozzi F., Van Nieuwenhove C. (2025). Fermented pomegranate juice enriched with pomegranate seed oil ameliorates metabolic disorders associated with a high-fat diet in C57BL/6 mice. Food Chem..

[39] Wei Z., Wang J., Wang Y., Wang C., Liu X., Han Z., Fu Y., Zhang N. (2019). Effects of neutrophil extracellular traps on bovine mammary epithelial cells in vitro. Front. Immunol..

[40] Zhou Y., Wang B., Wang Q., Tang L., Zou P., Zeng Z., Zhang H., Gong L., Li W. (2021). Protective Effects of *Lactobacillus plantarum* Lac16 on Clostridium perfringens Infection-Associated Injury in IPEC-J2 Cells. Int. J. Mol. Sci..

[41] Rainard P., Foucrasm G. (2018). A Critical Appraisal of Probiotics for Mastitis Control. Front. Vet. Sci..

[42] Yu Q., Xu C., Wang M., Zhu J., Yu L., Yang Z., Liu S., Gao X. (2022). The preventive and therapeutic effects of probiotics on mastitis: A systematic review and meta-analysis. PLoS ONE.

[43] Petzl W., Zerbe H., Günther J., Seyfert H.M., Hussen J., Schuberth H.J. (2018). Pathogen-specific responses in the bovine udder. Models and immunoprophylactic concepts. Res. Vet. Sci..

[44] Page M.J., McKenzie J.E., Bossuyt P.M., Boutron I., Hoffmann T.C., Mulrow C.D., Shamseer L., Tetzlaff J.M.., Akl E.A., Brennan S.E. (2021). The PRISMA 2020 statement: An updated guideline for reporting systematic reviews. BMJ.

