# Supplementary figures and images for: Probiotic Characterization of Lactiplantibacillus paraplantarum SDN1.2 and Its Anti-Inflammatory Effect on Klebsiella pneumoniae-Infected Mammary Glands
Source: Vet Sci. 2025 Apr 1;12(4):323. doi: 10.3390/vetsci12040323 (PMC12031044; doi:10.3390/vetsci12040323)

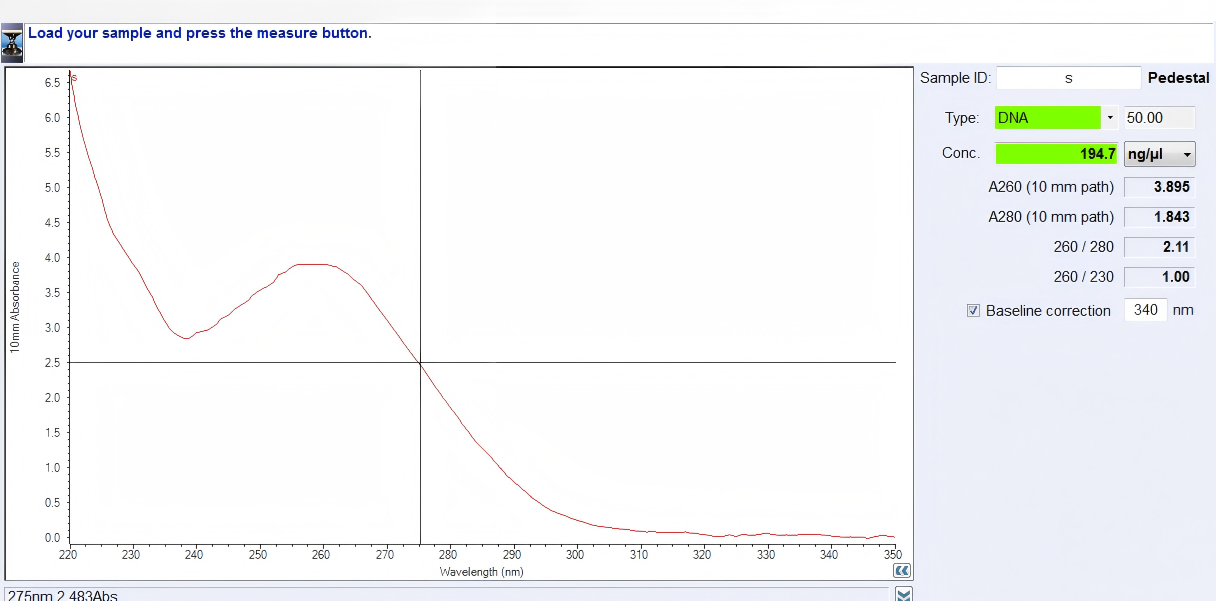


**Supplementary Data S2.** Nanodrop curve.

Supplement: Supplementary file 1 [file vetsci-12-00323-s001.zip › Supplementary Data S2.docx]
